# Trapping of Rift Valley Fever (RVF) vectors using Light Emitting Diode (LED) CDC traps in two arboviral disease hot spots in Kenya

**DOI:** 10.1186/1756-3305-5-94

**Published:** 2012-05-19

**Authors:** David P Tchouassi, Rosemary Sang, Catherine L Sole, Armanda DS Bastos, Lee W Cohnstaedt, Baldwyn Torto

**Affiliations:** 1 International Centre of Insect Physiology and Ecology, Nairobi, Kenya; 2 Department of Zoology and Entomology, University of Pretoria, Pretoria, South Africa; 3 Centre for Virus Research, Kenya Medical Research Institute, Nairobi, Kenya; 4 United States Department of Agriculture, Center for Grain and Animal Health Research, Agricultural Research Service, Manhattan, KS, USA

**Keywords:** Mosquito surveillance, Rift Valley Fever vectors, Light-emitting diodes, Light trap, Kenya

## Abstract

**Background:**

Mosquitoes’ response to artificial lights including color has been exploited in trap designs for improved sampling of mosquito vectors. Earlier studies suggest that mosquitoes are attracted to specific wavelengths of light and thus the need to refine techniques to increase mosquito captures following the development of super-bright light-emitting diodes (LEDs) which emit narrow wavelengths of light or very specific colors. Therefore, we investigated if LEDs can be effective substitutes for incandescent lamps used in CDC light traps for mosquito surveillance, and if so, determine the best color for attraction of important Rift Valley Fever (RFV) vectors.

**Methods:**

The efficiency of selected colored LED CDC light traps (red, green, blue, violet, combination of blue-green-red (BGR)) to sample RVF vectors was evaluated relative to incandescent light (as control) in a CDC light trap in two RVF hotspots (Marigat and Ijara districts) in Kenya. In field experiments, traps were baited with dry ice and captures evaluated for *Aedes tricholabis*, *Ae. mcintoshi*, *Ae. ochraceus*, *Mansonia uniformis*, *Mn. africana* and *Culex pipiens*, following Latin square design with days as replicates. Daily mosquito counts per treatment were analyzed using a generalized linear model with Negative Binomial error structure and log link using R. The incidence rate ratios (IRR) that mosquito species chose other treatments instead of the control, were estimated.

**Results:**

Seasonal preference of *Ae.mcintoshi* and *Ae. ochraceus* at Ijara was evident with a bias towards BGR and blue traps respectively in one trapping period but this pattern waned during another period at same site with significantly low numbers recorded in all colored traps except blue relative to the control. Overall results showed that higher captures of all species were recorded in control traps compared to the other LED traps (IRR < 1) although only significantly different from red and violet.

**Conclusion:**

Based on our trapping design and color, none of the LEDs outcompeted the standard incandescent light. The data however provides preliminary evidence that a preference might exist for some of these mosquito species based on observed differential attraction to these light colors requiring future studies to compare reflected versus transmitted light and the incorporation of colored light of varying intensities.

## Background

Mosquitoes are responsible for the transmission of several arboviral pathogens such as Rift Valley Fever virus (RVFv), which is associated with periodic outbreaks in domestic animals and humans in Africa and the Arabian Peninsula [1,2]. Early detection of the vectors and this pathogen is essential to reduce disease risk to humans and animals. Currently, the detection and monitoring of mosquitoes, is performed primarily using Centers for Disease Control and Prevention (CDC) light traps with incandescent bulbs, which are considered the industry standard for mosquito surveillance. However, improving mosquito-based arbovirus surveillance by increasing trap captures remains a priority to maximize viral detection probability especially during the inter-epidemic period (IEP) characterized by low vector population density and sporadic transmission foci.

The impact of the visual cues provided by the incandescent light used in the CDC light trap is important to trapping effectiveness. Earlier studies suggest that insects generally see and show preferences for three specific colors—ultraviolet (UV), blue, and green [3,4]. As such the incandescent light bulb currently used in mosquito surveillance may have the unintended effect of repelling some mosquito species, and may poorly target them [5] as it emits most strongly in the infrared spectra and weakly in the visible light spectra of blue, green, and red.

Improved trapping of mosquitoes has been achieved by determining mosquito responses to the color and intensity of light sources [4,6]. Previous studies have found that mosquitoes are attracted preferentially to specific wavelengths of light [7,8]. With advances in lighting technology, the super-bright light-emitting diodes (LEDs) have recently been developed which can be selected to emit a narrow bandwidth or specific color [9]. This configuration has been shown to work particularly well in enhancing trap catches of disease vectors and thus the need to refine techniques to increase mosquito captures by using more precise light sources.

Similar studies on preferential attraction to specific wavelengths of light have been reported in phlebotomine sand flies [10-12] and *Culicoides* flies [13]. In addition, observed distinct color and pattern preferences employed in trapping technology has been reported in tabanids [14,15], *Stomoxys* spp [16,17] and tsetse flies [17-19].

So far there has been no published work pertaining to the evaluation of colored LEDs for improved captures of field populations of mosquitoes in Disease Endemic Countries (DECs) in Africa. In an effort to develop a highly effective visual target for improved surveillance of different arboviral disease vectors, our goal was to determine whether LEDs can serve as effective substitutes for incandescent lamps used in the standard CDC mosquito traps for mosquito surveillance, and if so, to determine the best color for these arboviral disease vectors.

## Methods

### Study sites

The study sites were Ijara and Marigat districts, which are ecologically distinct and are hot spots for RVF activities in Kenya. In Ijara district located in North Eastern Province of the country, trapping experiments were conducted in two major communities: Sangailu and Kotile. The entire area is semi-arid and normally has two rainy seasons a year: the short rains between October and December and the long rains in March and April. The area is located at an altitude of about 100 m above-sea-level (asl) and typical annual rainfall averages between 300 to 500 mm. The people in North Eastern Province are ethnically nearly all Somali pastoralists. Vegetation predominantly consists of shrubs and acacia bushes, while livestock includes cattle, goats, sheep, camels, and donkeys. Livelihoods are primarily dependent on livestock.

**Figure 1:**
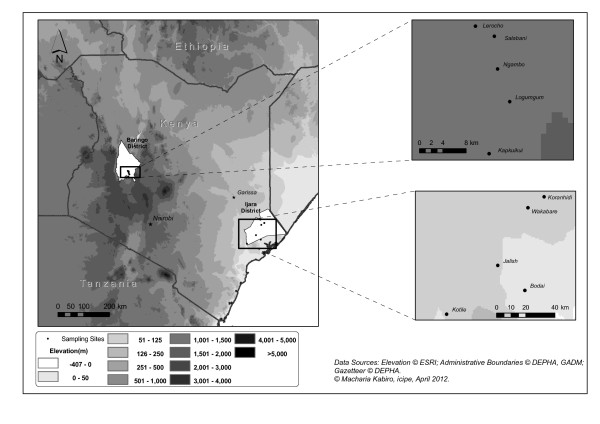
Mapping showing trapping sites in Kenya.

In Marigat District located in the Rift Valley Province of the country, trapping experiments were conducted in surrounding villages/communities namely N’gambo, Salabani, Bogoria and Sirata (Figure 1). The vegetation in the low lying arid part of the district consists of northern Acacia-Commiphora bushlands and thickets and has experienced severe land degradation caused by uncontrolled grazing. The local inhabitants mainly agro pastoralists, subsist mainly on limited crop production and livestock rearing. This area located around 3200 m asl receives annual rainfall ranging from 300 to 700 mm, with daily temperature variation between 16 and 42°C.

### Study design

Trials in Ijara area were run in December 2010 and May-June 2011 which coincided with peak and mild rainy season respectively to take advantage of peak mosquito populations. Experiments in Marigat area were conducted between July and September 2011 when there are rains to ascertain availability of mosquitoes. Mosquito captures in the BioQuip® LED CDC trap with different color platforms (part number, wavelength) of blue (2770B430, 430 nm), green (2770 G570, 570 nm), red (2770R660, 660 nm), violet (2770UV, 390 nm) BGR –were compared against a 1.5 watt incandescent light (control) in a standard CDC light trap (John Hock). Each LED assayed had 8 LEDs of the same color (arranged in a circular alignment) to provide 360-degree coverage in the horizontal plane with each LED having a viewing angle of 45 degrees. One array consisted of the combinations of several of different-colored LEDS and contained three green, 3 blue and two red LEDS (BGR). Super bright LED arrays typically produce 1–2 watts although non-superbright ultraviolet array produce about 800 mW. The incandescent bulb has a typical spectrum of an incandescent bulb with 95% of the energy emitted as heat. With an inter-trap distance of at least 40 m, all traps set following a Latin square design with days as replicates were activated 30 min before sunset and collected between 6:30–7:00, shortly after sunrise. All traps were baited with CO_2_ supplied in the form of dry ice to maximize collections.

### Data analyses

Daily count of each mosquito species recorded in the various trap treatments were analyzed using a generalized linear model with Negative Binomial error structure and log link using R 2.11.0 software [20]. Using the treatment incandescent light (control) as the reference category, the incidence rate ratios (IRR) that mosquito species chose other LED treatments colors instead of the control were estimated. The IRR for the control is 1 (unity) and values above this indicates better performance and values below under performance of the treatments relative to the control. Observed differences in the abundance and composition of mosquito species during the different trapping periods and districts/sites were analyzed independently of trapping period and districts/sites. Analyses were limited only to mosquito species that occurred in significant numbers to allow for discrimination across the different trap treatments. In Ijara, analysis was limited to flood water mosquitoes which are primary vectors of Rift Valley Fever (RVF) namely *Ae. mcintoshiAe. tricholabisAe. ochraceus* and/or *Cx. pipiens* sl (secondary vector) which were abundant at these sites but completely absent or occurred in extremely low numbers at Marigat except for *Cx. pipiens* sl. Data was analyzed in Marigat for *Mn. uniformisMn. africana* and *Cx. pipiens* sl., all secondary RVF vectors.

## Results

*Aedes tricholabis* Higher captures of this species were recorded in control traps compared to other treatments (Table 1). Overall, order of performance was control > BGR > violet then followed by the other colors with red performing least. There was a highly significant effect of treatments on this species at Ijara both during the experimental period of December 2010 (χ^2^ = 154.913, d.f. = 5, p = 0.003) and May-June 2011 (χ^2^ = 74.893, d.f. = 5, p = 0.0000). When compared to the control trap during December 2010, significantly fewer captures for this species were recorded in blue, green and red colors. Equally, higher captures were recorded in the control relative to BGR and violet colors, although the differences were not significantly different (Table 2). However, analysis of the results during the low density period of May 2011 revealed significantly lower numbers of this species were recorded in all the colored traps compared to the control incandescent light (Table 3).

**Table 1:** Composition of RVF mosquito species collected in traps with different lights at three time intervals and two locations

Experimental period	N	Species	Treatment					
			Control (incandescent light)	BGR	Blue	Green	Red	Violet
Ijara December 2010	14	*Ae. tricholabis*	3464	2245	1141	1338	895	1395
		*Ae.mcintoshi*	198	255	137	119	184	121
		*Ae.ochraceus*	734	732	913	789	582	192
IjaraMay- June 2011	11	*Ae. tricholabis*	2755	969	604	314	267	781
		*Ae.mcintoshi*	196	84	108	64	38	43
		*Ae.ochraceus*	162	54	61	23	31	40
		*Cx. pipiens*	244	86	81	26	26	78
Marigat July-September 2011	17	*Mn. uniformis*	1195	941	853	747	580	566
		*Mn. africana*	1134	504	538	531	464	438
		*Cx. pipiens*	682	385	377	242	227	331

N = No. of replicates.

**Table 2:** Comparisons of colored LED collections relative to the control (incandescent light) for trapping experiment at Ijara district, December 2010

Vector species	Treatment comparison relative to the control	IRR (95% CI)	P-value
*Ae. tricholabis*	BGR	0.78 (0.36–1.67)	0.511
	blue	0.37 (0.17–0.81)	0.0113 *
	green	0.41 (0.19–0.89)	0.0220 *
	red	0.44 (0.20–0.97)	0.0336 *
	violet	0.47 (0.22–1.02)	0.0539 .
*Ae. mcintoshi*	BGR	1.44 (0.60–3.44)	0.4031
	blue	0.46 (0.19–1.13)	0.0878 .
	green	0.63 (0.26–1.55)	0.3093
	red	0.72 (0.30–1.74)	0.4552
	violet	0.33 (0.13–0.83)	0.0182 *
*Ae. ochraceus*	BGR	0.99 (0.23–4.27)	0.16989
	blue	1.24 (0.29–5.32)	0.22918
	green	1.07 (0.25–4.60)	0.22939
	red	0.79 (0.19–3.39)	0.11809
	violet	0.27 (0.12–0.57)	0.000384 ***

Estimated incidence rate ratio (IRR); Confidence interval (CI) and corresponding P-values; Asterisks indicate that the index is significantly different from unity at the P,0.05 (*), P,0.01 (**), P,0.001 (***) levels of probability.

**Table 3:** Comparisons of colored LED collections relative to the control (incandescent light) for trapping experiment at Ijara district, May-June 2011

Vector species	Treatment comparison relative to the control	IRR (95% CI)	P-value
*Ae. tricholabis*	BGR	0.36 (0.17–0.78)	0.009095 **
	blue	0.20 (0.09–0.45)	4.76e–05 ***
	green	0.11 (0.05–0.25)	2.83e–08 ***
	red	0.10 (0.05–0.22)	5.61e–09 ***
	violet	0.27 (0.12–0.59)	0.000737 ***
*Ae. mcintoshi*	BGR	0.45 (0.22–0.92)	0.025934 *
	blue	0.56 (0.28–1.13)	0.10305
	green	0.33 (0.16–0.68)	0.002586 **
	red	0.19 (0.09–0.40)	1.41e–05 ***
	violet	0.24(0.11–0.50)	0.000115 ***
*Ae. ochraceus*	BGR	0.35 (0.16–0.77)	0.009581 **
	blue	0.44 (0.20–0.98)	0.042136 *
	green	0.18 (0.08–0.44)	8.28e–05 ***
	red	0.20 (0.08–0.45)	0.000141 ***
	violet	0.25 (0.11–0.56)	0.000881 ***
*Cx. pipiens*	BGR	0.41 (0.15–1.10)	0.0567 .
	blue	0.34 (0.13–0.86)	0.0214 *
	green	0.11 (0.04–0.30)	1.08e–05 ***
	red	0.11 (0.04–0.31)	1.26e–05 ***
	violet	0.35 (0.13–0.91)	0.0253 *

Estimated incidence rate ratio (IRR); Confidence interval (CI) and corresponding P-values; Asterisks indicate that the index is significantly different from unity at the P, 0.05 (*), P, 0.01 (**), P, 0.001 (***) levels of probability.

*Aedes mcintoshi* A similar significant effect of treatments on this species capture during December 2010 (χ^2^ = 174.128, d.f. = 5, p = 0.0492) was observed but only after taking into account the effect of replicates and site (Kotile and Sangailu) but with a marked effect of the treatments on captures during May-June 2011 (χ^2^ = 76.765, d.f. = 5, p = 0000). In December 2010, apart of BGR treatment which recorded a 44% increase in captures compared to the control [IRR = 1.44, CI (0.60–3.44)] all the other treatments recorded lower captures relative to the control (IRR < 1) which were however only significantly different from those recorded in violet (Table 2). Surprisingly, this pattern dwindled during the low period of mosquito population density in May 2011 where significantly lower capture numbers were recorded in all the colored light treatments when compared to the control (IRR < 1) except blue (Table 2).

*Aedes ochraceus* An analogous effect of treatment on the captures of this species was evident during both trapping periods of December 2010 (χ^2^ = 415.93, d.f. = 5, p = 0.0002) and May-June 2011 (χ^2^ = 90.398, d.f. = 5, p = 000000). In December 2010, a slight preference for blue-green colors was apparent with increases in captures of 24 and 7% recorded in blue [IRR = 1.24, CI (0.29–5.32)] and green IRR = 1.07, CI (0.25–4.60)] colored traps respectively compared to the control which were not significantly different. During this period, fewer *Ae. ochraceus* captures were recorded in the remaining colored traps relative to the control (IRR < 1) which was only significantly different from violet (Table 2). The trend dwindled during the trial in May 2011 where significantly fewer were observed in all the colored traps compared to the control incandescent light (Table 3).

*Culex pipiens* sl Treatment significant effect were observed both during May-June trial at Ijara 2011 (χ^2^ = 75.284, d.f. = 5, p = 0.00001) and Marigat (χ^2^ = 118.10, d.f. = 5, p = 0.02064) on the captures of this species. Significantly fewer were captured in all the colored traps compared to incandescent light (IRR < 1) except for BGR light in Ijara 2011 and BGR and blue at Marigat (Tables 3 and 4).

**Table 4:** Comparisons of colored LED collections relative to the control (incandescent light) for trapping experiment at Marigat district, July-September 2011

Vector species	Treatment comparison relative to the control	IRR (95% CI)	P-value
*Mn. uniformis*	BGR	0.79 (0.41–1.53)	0.4757
	blue	0.71 (0.37–1.38)	0.3145
	green	0.63 (0.32–1.21)	0.1613
	red	0.49 (0.25–0.94)	0.0314 *
	violet	0.47 (0.24–0.92)	0.0262 *
*Mn. africana*	BGR	0.44 (0.21–0.95)	0.0340 *
	blue	0.47 (0.22–1.02)	0.0512 .
	green	0.47 (0.22–0.99)	0.0472 *
	red	0.41 (0.19–0.87)	0.0196 *
	violet	0.39 (0.18–0.82)	0.0130 *
*Cx. pipiens*	BGR	0.56 (0.28–1.13)	0.10536
	blue	0.55 (0.27–1.11)	0.09325 .
	green	0.35 (0.18–0.71)	0.00354 **
	red	0.33 (0.16–0.67)	0.00198 **
	violet	0.49 (0.24–0.98)	0.04096 *

Estimated incidence rate ratio (IRR); Confidence interval (CI) and corresponding P-values; Asterisks indicate that the index is significantly different from unity at the P, 0.05 (*), P, 0.01 (**), P, 0.001 (***) levels of probability.

*Mansonia uniformis* There was no overall significant effect of treatments on the species trap captures across treatment replicates (χ^2^ = 116.05, d.f. = 5, p = 0.1896). Although fewer were captured in light traps compared to the control incandescent light, it was only significantly so for violet and red light but not for BGR, blue and green (Table 4).

*Mansonia africana* and (χ^2^ = 76.765, d.f. = 5, p = 0.0000) Analogous response patterns were observed for *Mn. africana* with no overall significant effect of treatments on the species trap captures across treatment replicates (χ^2^ = 118.70, d.f. = 5, p = 0.08368). However, captures were all significantly less in all colored lights relative to incandescent except blue (Table 4).

## Discussion

The observed variation in trap captures recorded in the different colored configurations suggests that mosquito species vary in attractiveness to light-baited traps [21,22]. As such it is logical to expect that individual species wavelength preference will vary although such behavioral wavelength preferences may or may not correspond to spectral sensitivities [8].

Following our study design, the incandescent light recorded an overall higher capture of mosquitoes compared to any other LED colored traps (red, blue, green, violet, BGR). This was followed by BGR, blue, green, violet and red in the order of performance for most of the mosquito species examined. The results of field trials by Burkett et al. [4] with LED-modified CDC traps observed color preferences for some species of *AnophelesCulexCulisetaOchlerotatus*, and *Psorophora*. With a significant effect of light color on capture numbers, blue or green light was particularly preferred in most instances, with incandescent light most often performing nearly as well as blue and green light and generally better than red, orange, or yellow light. A similar order of effectiveness of green > incandescent > blue > red light, in trapping mosquitoes from three genera (*AnophelesCulex* and *Aedes*) was reported by Hoel et al. [11]. This contrasts with our findings where incandescent light proved to be superior in a majority of instances compared to the LED colors used in our experiment although in terms of performance were followed by BGR or blue and green in this order. This difference might be related to the lighting design used in our experiment. The LEDs used in our design produce only transmitted light (direct line of sight) at very specific frequencies as opposed to reflected light (off of aluminum rain shields) used in the abovementioned previous studies. Transmitted light might not be as scattered as reflected light thereby reducing visual contrast and target size [11].

A related work by Burkett comparing blue and green light LED-modified CDC traps to incandescent light traps in north Florida demonstrated that, with the exception of *Culex* (*Melanoconion*) spp., mosquitoes showed no preferences between incandescent, blue, or green light as either transmitted light or reflected light (Doug Burkett, personal communication)(cf: [11]. In this regard, the lack of consistency in trap performance to colored light remains a challenge. Microhabitat/ecological and seasonal differences might interplay as evident in our data where a clear bias for BGR and blue lights was observed for *Ae. mcintoshi* and *Ae. ochraceus* respectively during one trapping season but dwindling effect recorded in another season. Although the reason for this is unclear; perhaps, environmental changes such as dust storms or vegetation changes could lead to reduced brightness of the LEDs and therefore attraction to mosquitoes. As such, the intensity of the light produced by the LEDs needs to be considered. The traps were all baited with dry ice to enhance trap captures. Using CO_2_ is important because it is a long range attractant and the light color is a short range attractant. Therefore, to bring in statistically significant numbers of mosquitoes to compare trap captures, a long range attractant is needed to bring the mosquitoes into closer proximity at which time their photo attraction will supersede the chemo attraction. A standardized amount of dry ice (1 Kg) was used for this purpose and it is unlikely that the addition of CO_2_ may have affected the trapping experiments.

In a previous study using several colored light bulbs of different intensities to capture mosquitoes (*Anopheles* and *Aedes* species), Barr et al. [23] determined that color had little effect on trap capture and that light intensity played a significant role with higher intensity lights (100 W lamps) being more attractive than lower intensity light (60 W and 25 W lamps). Similarly, Breyev [24] reported significant attraction of *Ae. vexans* with one 220 W mercury lamp than with two 109 W incandescent lamps. However, evaluation of six different colors (white, yellow, green, orange, blue, and red) of varying intensities on mosquito captures by Ali et al. [25] found that five predominate species (*Psorophora columbiaePs. ciliataCulex salinariusCx. nigripalpus*, and *Cx. erraticus*) were much more strongly affected by color than by light intensity. A similar pattern was established by Gjullin et al. [26] who found no evidence of importance of light intensity over color for mosquito attraction. The above results therefore suggest confounding findings regarding the relative importance of light color and intensity in mosquito attraction.

Stacking 2 LED lighting chips (16 LED bulbs) can provide an equal measure of light intensity of each colored LED trap platform compared to the incandescent light used in our experiment (Cohnstaedt, personal communication) although this was not possible with the trap designs we used. However with sufficient evidence that light color and intensity affect trap attractiveness to mosquitoes, and that mosquito species appear able to discern color and in cases prefer some colors to others [27-29] it may be worthwhile to consider in future studies, colored light of varying intensities.

Mosquitoes response to artificial light in many field and laboratory studies have reported a dominant spectral sensitivity to light in the ultraviolet-blue and green light and incandescent light spectrum [4,30,31]. Although recorded lower captures compared to incandescent, BGR and Blue colors performed better than violet and red for most species including *Mn. uniformisMn. africana* and *Cx. pipiens*. This concurs with findings that blue and green light is often more attractive than light in the yellow-orange and red regions of the visible spectrum. Many insects are insensitive to red spectrum frequencies as noted by Breyev [24]. This may account for lower captures recorded in the red colored light compared to the others for most of the mosquito species. This observation however, may not pertain to sandfly species. In fact Hoel et al. [11] in a field study in southern Egypt found that over half (55.13%) of all sand flies were collected from red light traps with significantly more recorded than in blue, green, or incandescent light traps.

Our data suggest clearly that irrespective of trapping period, most of the mosquito species were far more attracted to multi-spectrum light (incandescent light) as compared to monochromatic light. It is possible that the high intensity incandescent light was favored over the lower intensity monochromatic lights due to superior luminosity/intensity even though further studies are required to ascertain this.

## Conclusions

Based on color alone, the data suggest that none of the colored lights is an effective substitute for standard incandescent light currently being used for surveillance of mosquitoes. This work notwithstanding presents preliminary evidence that a preference might exist for some of these mosquito species to light colors, therefore more studies to determine optimal color preferences of medically important mosquitoes are desirable and worth evaluating across a range of microhabitats in diverse ecologies. Future studies should consider comparing reflected versus transmitted light and incorporation of colored light of varying intensities.

## Competing interests

The authors declare that they have no competing interests.

## Authors’ contributions

DPT RS BT conceived and designed experiments. DPT conducted the experimental work. DPT analyzed the data. DPT RS CLS ADSB LWC BT contributed to the manuscript. All authors approved the final version for submission.
